# Neuroinflammation and ER-stress are key mechanisms of acute bilirubin toxicity and hearing loss in a mouse model

**DOI:** 10.1371/journal.pone.0201022

**Published:** 2018-08-14

**Authors:** Emanuele Schiavon, Joshua L. Smalley, Sherylanne Newton, Nigel H. Greig, Ian D. Forsythe

**Affiliations:** 1 Department Neuroscience, Psychology & Behaviour, University of Leicester, Leicester, Leicestershire, United Kingdom; 2 Translational Gerontology Branch, Intramural Research Program, National Institute on Aging, NIH, Baltimore, MD, United States of America; Universidad de Salamanca, SPAIN

## Abstract

Hyperbilirubinemia (jaundice) is caused by raised levels of unconjugated bilirubin in the blood. When severe, susceptible brain regions including the cerebellum and auditory brainstem are damaged causing neurological sequelae such as ataxia, hearing loss and kernicterus. The mechanism(s) by which bilirubin exerts its toxic effect have not been completely understood to date. In this study we investigated the acute mechanisms by which bilirubin causes the neurotoxicity that contributes to hearing loss. We developed a novel mouse model that exhibits the neurological features seen in human Bilirubin-Induced Neurological Dysfunction (BIND) syndrome that we assessed with a behavioural score and auditory brainstem responses (ABR). Guided by initial experiments applying bilirubin to cultured cells *in vitro*, we performed whole genome gene expression measurements on mouse brain tissue (cerebellum and auditory brainstem) following bilirubin exposure to gain mechanistic insights into biochemical processes affected, and investigated further using immunoblotting. We then compared the gene changes induced by bilirubin to bacterial lipopolysaccharide (LPS), a well characterized inducer of neuroinflammation, to assess the degree of similarity between them. Finally, we examined the extent to which genetic perturbation of inflammation and both known and novel anti-inflammatory drugs could protect hearing from bilirubin-induced toxicity. The *in vitro* results indicated that bilirubin induces changes in gene expression consistent with endoplasmic reticulum (ER) stress and activation of the unfolded protein response (UPR). These gene changes were similar to the gene expression signature of thapsigargin–a known ER stress inducer. It also induced gene expression changes associated with inflammation and NF-κB activation. The *in vivo* model showed behavioural impairment and a raised auditory threshold. Whole genome gene expression analysis confirmed inflammation as a key mechanism of bilirubin neurotoxicity in the auditory pathway and shared gene expression hallmarks induced by exposure to bacterial lipopolysaccharide (LPS) a well-characterized inducer of neuroinflammation. Interestingly, bilirubin caused more severe damage to the auditory system than LPS in this model, but consistent with our hypothesis of neuroinflammation being a primary part of bilirubin toxicity, the hearing loss was protected by perturbing the inflammatory response. This was carried out genetically using lipocalin-2 (LCN2)-null mice, which is an inflammatory cytokine highly upregulated in response to bilirubin. Finally, we tested known and novel anti-inflammatory compounds (interfering with NF-κB and TNFα signalling), and also demonstrated protection of the auditory system from bilirubin toxicity. We have developed a novel, reversible, model for jaundice that shows movement impairment and auditory loss consistent with human symptoms. We used this model to establish ER-stress and inflammation as major contributors to bilirubin toxicity. Because of the rapid and reversible onset of toxicity in this novel model it represents a system to screen therapeutic compounds. We have demonstrated this by targeting inflammation genetically and with anti-inflammatory small molecules that offered protection against bilirubin toxicity. This also suggests that anti-inflammatory drugs could be of therapeutic use in hyperbilirubinemia.

## Introduction

Bilirubin is a product of haem metabolism and excessive accumulation can occur following haemolysis or compromised excretion from the liver into the bile. Circulating bilirubin is generally bound to serum albumin, but high levels of free-bilirubin can result in passage across the Blood-Brain-Barrier (BBB), causing neurotoxicity and damage to the central nervous system. Neurotoxicity in jaundice is noted in the cerebellum, basal ganglia and brainstem auditory nuclei [1–3]. Although mild Jaundice in neonates is relatively common (affecting 60% of premature new-borns) and considered a benign condition, 8–9% of those cases are exposed to high bilirubin levels where there is the risk of developing permanent brain damage and/or hearing loss. There is also evidence to suggest a delayed risk of hearing loss in humans, occurring years after an acute exposure of hyperbilirubinemia [4].

The molecular mechanism(s) of bilirubin toxicity in the brain are unknown. Bilirubin causes gross pathological changes, including demyelination [5–10] and cerebellar hypoplasia [11–14]. Bilirubin can perturb calcium homeostasis [15–17] and change ER structure *in vivo* [11,18], while ER stress and the unfolded protein response (UPR) have been observed *in vitro*; along with oxidative stress and inflammation [19]. Existing models for hyperbilirubinemia, such as the Gunn rat (which lacks glucoronyltransferase and so compromises the hepatic route of bilirubin excretion) show elevated bilirubin from early development, which persists throughout the lifetime of the animal. This is a good model for chronic hyperbilirubinemia [20–24], but there is a need to investigate the acute toxicity of bilirubin, as this represents the early stages of jaundice that occurs in humans and particularly new-born babies.

In this study, we used several approaches to investigate the acute mechanisms by which bilirubin induces toxicity: first we observed the global gene expression changes induced by the application of bilirubin in a human cell line (SHSY5Y). This provided a mechanistic understanding of bilirubin toxicity in an isolated system. Using the Connectivity Map (cMap), a microarray data analysis technique, [25–28] also allowed parallels to be drawn between bilirubin and other better characterized chemicals. Second, we developed and validated an *in vivo* mouse model of acute bilirubin toxicity in the brain of young mice, which exhibit the ataxic behaviour and hearing deficits reported in humans and other animal models [4,24]. Third, we measured whole genome gene expression from the cerebellum and auditory brainstem of mice exhibiting neurological dysfunction following exposure to bilirubin. We found bilirubin exposure induced several inflammatory markers similar to LPS—a canonical neuroinflammatory agent [29]. We then investigated how interfering with this inflammatory response either pharmacologically or genetically affected the bilirubin toxicity in the brain.

Here we observed that the induction of endoplasmic reticulum (ER) stress and NF-κB-mediated neuroinflammation, correlated with the neurological deficits observed in our novel model of hyperbilirubinemia. ER stress is a process triggered by the accumulation of misfolded proteins in the ER. Upon detection, IRE1, PERK and ATF6 increase the expression of protein-folding chaperones such as BiP and DNAJB9, and reduce the protein load by attenuating translation via eIF2α [30,31]. If unresolved, then ER stress initiates apoptosis through the induction of CHOP and caspase activation [32]. ER stress has been identified in a number of pathological processes in the brain, including Alzheimer’s, Parkinson’s and Huntington’s disease [33]. The NF-κB pathway is also activated in response to cellular stress. Such stresses can include oxidative stress or exposure to antigens and can lead to a variety of immunological responses that attempt to clear the source of the response. During activation, IKK phosphorylates IkB; the endogenous inhibitor of NF-κB, which is subsequently targeted for degradation. NF-κB then translocates to the nucleus and is involved in the transcription of a large number of pro-inflammatory cytokines and chemokines [34].

Here, we create a novel model of bilirubin toxicity and show that ER stress and NF-κB play a vital role in the toxicity of bilirubin. Further to this we show that the suppression of inflammation, using transgenic mice lacking LCN2, or by pharmacological interference, protects against bilirubin-induced hearing loss and points to new therapeutic strategies.

## Results

### Bilirubin activates the ER-stress and NF-κB pathways *in vitro*

**Fig 1:**
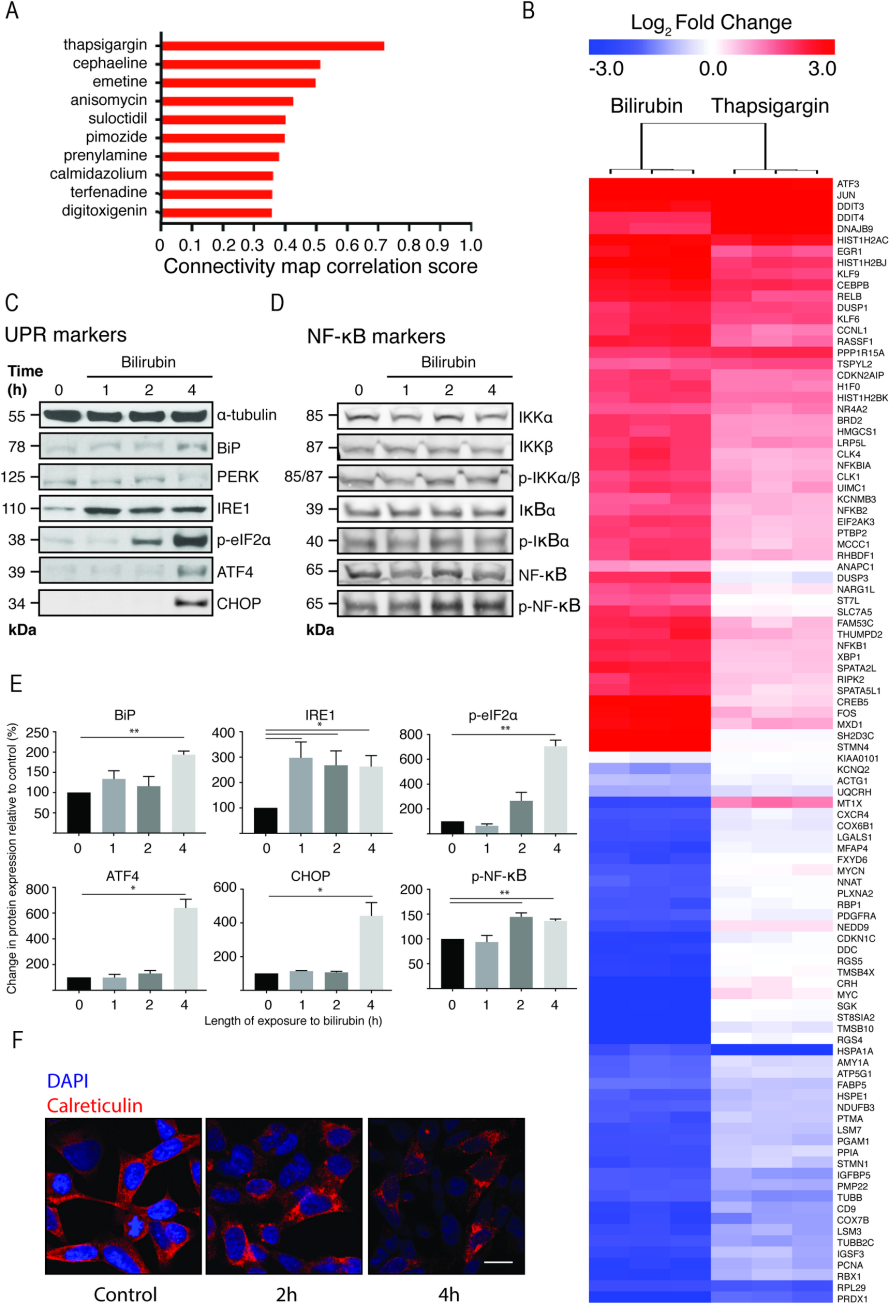
Bilirubin exposure activates ER stress and NFκB pathways in SHSY5Y cells. SHSY5Y cells were exposed to 50 μM bilirubin for 4h. Whole genome gene expression analysis was subsequently carried out. **A)** The gene expression pattern was compared to a compendium of 1600 chemical treatments using the cMap; the figure shows the correlation score for the top 10 chemicals, with bilirubin sharing greatest similarly to thapsigargin. **B)** The heatmap shows the correlation between the top 100 most changed genes in response to bilirubin or thapsigargin (n = 3). Samples and genes were clustered using the Euclidean distance algorithm. Up-regulated genes are shown in red and down-regulated genes in blue. The values represent log_2_ fold change compared to control samples. **C)** Representative images of immunoblots measuring the protein expression levels for ER stress marker proteins and **D)** NF-ĸB marker proteins (n = 3). **E)** Densitometry-based quantification of the immunoblots where significant changes in protein expression were detected by ANOVA (*p<0.05, **p<0.01). **F)** Representative images of morphological changes in the ER caused by application of 50μM bilirubin. The ER was visualised using an antibody for the ER membrane protein, calreticulin. The scale bar represents 10 μm.

We first investigated the mechanism by which bilirubin causes toxicity in a human neuronal cell line by measuring gene expression changes induced by bilirubin. We exposed SHSY5Y cells to 50 μM bilirubin for 4 hours. This is a high but sub-cytotoxic concentration of bilirubin that equates to 6.39 μM of unconjugated bilirubin (data not shown), followed by whole genome gene expression analysis. As an initial analysis the changes in gene expression were compared to changes induced by a panel of over 1600 chemicals using the Broad Institute’s Connectivity Map (cMap) [25]. We compared the top 100 most changed genes (selected by absolute fold change) following bilirubin treatment to the cMap database. This identified thapsigargin, a potent inhibitor of SERCA (sarco/endoplasmic reticulum calcium ATPase) in the endoplasmic reticulum, as the chemical producing the most ‘similar’ gene expression changes to bilirubin as shown by the high connectivity map correlation score (Fig 1A) with the comparison of the changes in mRNA expression (increased, red; decreased, blue) for bilirubin and thapsigargin shown in Fig 1B).

Thapsigargin is a sesquiterpine lactone known to induce both ER stress [30] and activation of NF-κB signalling [35]. Several chemicals identified here as generating similar gene expression profiles to bilirubin (pimozide, calmidazolium, terfenadine) also induce a non-canonical form of ER stress [36].

The most highly up-regulated gene cluster that correlated between bilirubin and thapsigargin include ATF3, JUN, DDIT3, DDIT4 and DNAJB9 (Fig 1B). These genes are well established transcriptional markers of ER stress and the UPR [36]. Three other transcriptional markers of ER stress and the UPR; CEBPB, DUSP1 and XBP1, were also induced by bilirubin, but these were less highly correlated with thapsigargin. Additionally, JUN, RELB, EGR1, NR4A2, NFKBIA, NFKB1, NFKB2, RIPK2, and FOS (Fig 1B) that are transcriptional products of active NF-κB [37], were up-regulated and positively correlated with bilirubin and thapsigargin. The NF-κB pathway is associated with the inflammatory response. Taken together, these data suggest that bilirubin induces the UPR and ER stress as well as the inflammatory response mediated by the NF-κB pathway *in vitro*.

To investigate this further we assayed the protein expression of markers for both the ER stress and NF-κB pathways by immunoblot (Fig 1C–1E). Following a 4h exposure to 50 μM bilirubin, SHSY5Y cells showed significantly increased expression of the chaperone protein BiP and IRE1 and decreased electrophoretic mobility of PERK, consistent with protein phosphorylation. Phosphorylated eIF2α (p- eIF2α) was detected at 2 hours, and heavily induced at 4 hours, along with ATF4 and CHOP. These observations indicate that several arms of the ER stress signalling pathway are activated by bilirubin. We also probed for several protein markers of the NF-κB signalling pathway. We observed no changes in phospho-IKK or phospho-IκB levels; however, phospho-NF-κB was significantly increased (Fig 1D and 1E) at 2 and 4 hrs, confirming activation of NF-κB, and consistent with triggering an inflammatory process.

In order to assess the ER morphology of SHSY5Y cells exposed to bilirubin we stained for the ER-marker protein calreticulin (Fig 1F). Control cells showed the ER evenly distributed through the soma cytoplasm. Exposure to 50 μM bilirubin caused ER fragmentation (Fig 1F, at 2h and 4h) and distinct clustering consistent with similar observations during ER stress [36, 38].

### Hearing loss and ataxia in a novel mouse model of acute hyperbilirubinemia

Characterization of bilirubin toxicity *in vivo* was achieved by developing a new acute model for hyperbilirubinemia in young CBA/Ca mice. A single intraperitoneal (IP) injection of bilirubin (accompanied by sulfodimetoxine to reduce serum binding, see methods) was sufficient to induce hyperbilirubinaemia and generate the behaviour and neurological symptoms correlated with jaundice in a dose-dependent manner *in vivo*, over a time course of 24 hrs.

**Fig 2:**
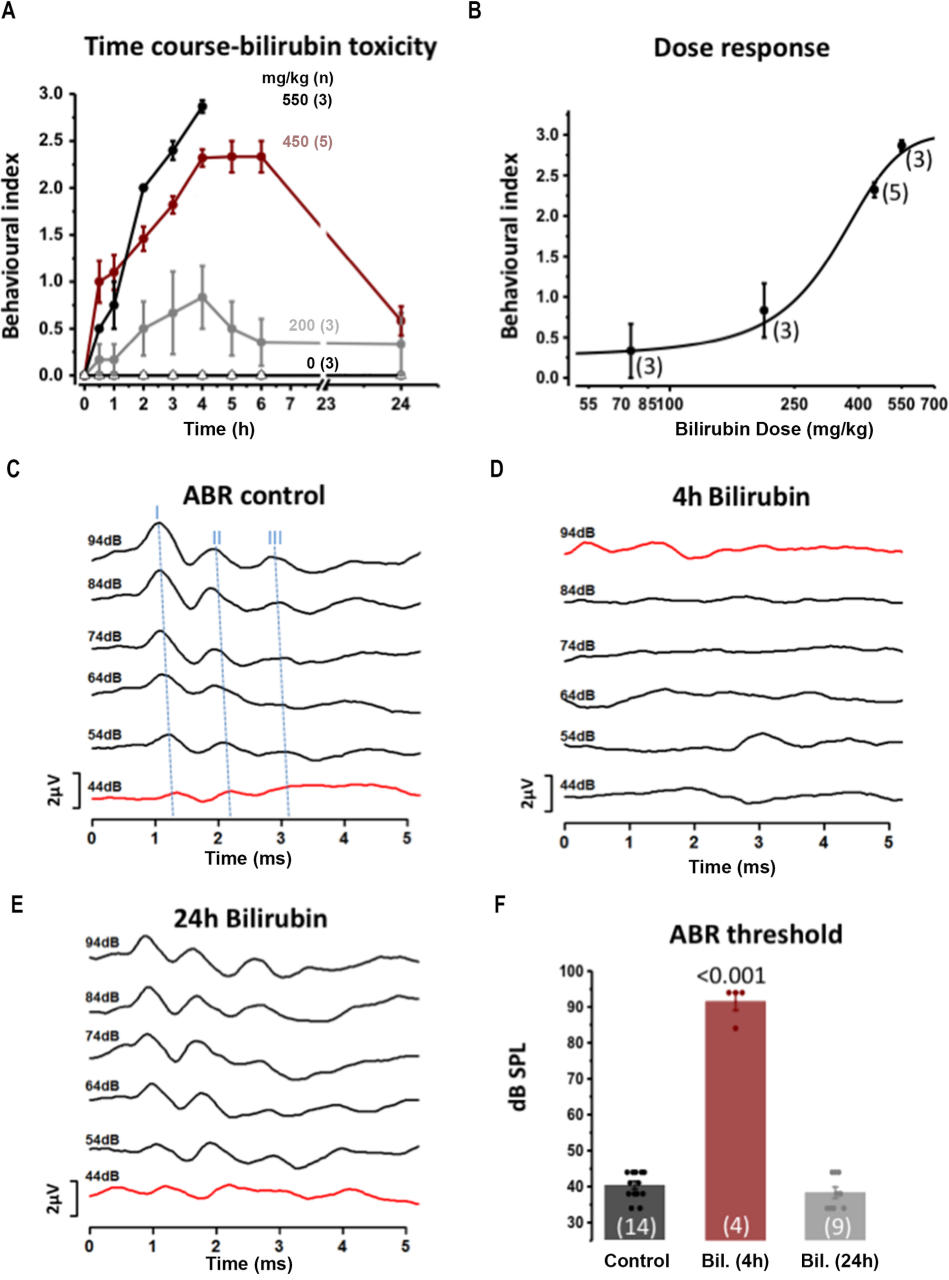
A mouse model for hyperbilirubinemia shows bilirubin toxicity on movement behavior and the auditory threshold. A behavioural index (0–3, see methods) was used to assay bilirubin effects on movement and wellbeing over a 24h period in mice. **A)** Plot of the behavioural index from the time of IP injection *in vivo* across a range of bilirubin doses (in presence of 300 mg/kg sulfadimethoxine). Filled black circles: 550mg/kg (n = 3) for 0-4h after injection; filled red circles: 450mg/kg (n = 5 for 0-6h and n = 24 24h); Filled grey circles: 200mg/kg (n = 3); Open triangles: Control 0mg/kg (n = 3) were injected with sulfadimethoxine, alone. **B)** The dose-response for bilirubin (75-550mg/kg) on the mouse behavior assayed using the Behavioural Index, 4h after bilirubin injection. The mean ± SEM (n = 3 to 5 animals) is fit to the Hill equation giving a half-maximal toxicity of 341±22 mg/kg. **C)** Hearing function across the same time-course as bilirubin exposure was assayed by measuring the ABR: control traces (before bilirubin exposure) are shown plotted against time and with increasing sound intensity from 44–94 dB SPL. Blue dashed lines mark the peak ABR waves I-II-III. **D)** IP injection of 450 mg/kg of bilirubin caused a near complete loss of the ABR **E)** which recovered after 24 h. **F)**. The ABR thresholds were measured at 4h or 24h after bilirubin injection (control is injection of sulfadimethoxine alone) = 40.1 ± 1.0 dB SPL, n = 14; bilirubin 4h = 91.5 ± 2.5 dB SPL, n = 4; bilirubin 24h = 38.2 ± 1.5 dB SPL, n = 9, ANOVA followed by Bonferroni P<0.001. Note that only one animal was tested at 4 and 24h, the other 8 animals were tested only at 24h, see methods).

We developed a behavioural index of well-being (see methods and video in additional files S1 File) and observed a dose-dependent increase in bilirubin toxicity following IP injection (Fig 2A). A dose of 75mg/kg bilirubin induced little or no movement abnormalities (behavioural index ± SEM = 0.33±0.33, n = 3 at 4h after injection, not shown in Fig 1A for clarity), while injection of 200 mg/kg reduced exploration relative to untreated littermates. The behavioural changes peaked at 4h after the injection (behavioural index 0.83±0.30, n = 3) and recovered within 6h, giving a mean behavioural index of 0.35±0.25 (Fig 2A, and see method for detail of behavioural index and well-being). IP injection of 450 mg/kg bilirubin induced a rapid onset and reproducible behavioural impairment that reached a plateau at 4-5h with a mean behavioural index score of 2.33±0.08, n = 5 (Fig 2A and a video in S1 File). After 24h, 24 of 31 animals (77.5%) had recovered to an average behavioural index of 0.58±0.15 (Fig 2A) and 22.5% died or were culled within 24h. The injection of 550 mg/kg of bilirubin induced rapid and severe toxicity. At 4h post injection the mean behavioural index was 2.86±0.06, n = 3 and the animals were culled at this time point, as they were moribund. The dose-response relationship (Fig 2B) showed increasing severity of behavioural impairment in response to increasing bilirubin doses at 4h post-injection. The experimental points (n = 3–5 animals) were fitted to a dose response curve (Hill equation) providing a dose of 341±22 mg/kg to obtain 50% of the maximal toxicity.

The 450 mg/kg dose of bilirubin was used to assess bilirubin-induced auditory impairment by measuring the ABR in these mice. Evoked ABRs were used to determine the extent to which bilirubin damaged the brainstem auditory nuclei, as previously reported in the Gunn rat model [20–23]. ABRs generated by a click stimulus (94 dB SPL) in CBA/Ca mice showed distinct waveforms of which the first and subsequent waves originate by synchronous firing of spiral ganglion neurons, the cochlear nucleus and the superior olivary complex (SOC), respectively (Fig 2C). The minimum sound intensity to evoke a response (threshold) was on average 40.1 ± 1.0 dB SPL (n = 14) in control animals (Fig 2C & 2F). Littermates injected with bilirubin (450 mg/kg) showed a reversible loss of normal ABR waveforms (Fig 2D), with auditory thresholds raised to 91.5 ± 2.5 dB. SPL (n = 4) 4h after the injection (Fig 2D & 2F), and recovery to control levels within 24h (threshold = 38.2 ± 1.5 dB SPL n = 9, Fig 2E & 2F) and 5 days (threshold = 38.1 ± 1.5 dB SPL, n = 6). ANOVA followed by Bonferroni p<0.001 confirmed that the mean of the ABR threshold 4h after the injection was significantly different from control and from the 24 h and 5 day recovery conditions. This mouse model is a valuable tool to investigate the mechanism(s) of bilirubin toxicity.

### Bilirubin-induces ER stress and NF-κB activation *in vivo*

Bilirubin induced ER stress and activation of the NF-κB pathway *in vitro*, therefore we tested whether bilirubin caused similar effects in the mouse model. In an initial experiment mRNA from broad regions of the brainstem and cerebellum were compared, prior to a focus on subdivisions of the brainstem auditory pathway, including the Cochlear Nucleus (CN) and the Medial Nuclei of the Trapezoid Body (MNTB). Whole genome gene expression analysis was conducted at 4 or 24 hours after mice were exposed to bilirubin (IP 450mg/kg) and compared to sham control mice which received no bilirubin (n = 4 for each condition).

**Fig 3:**
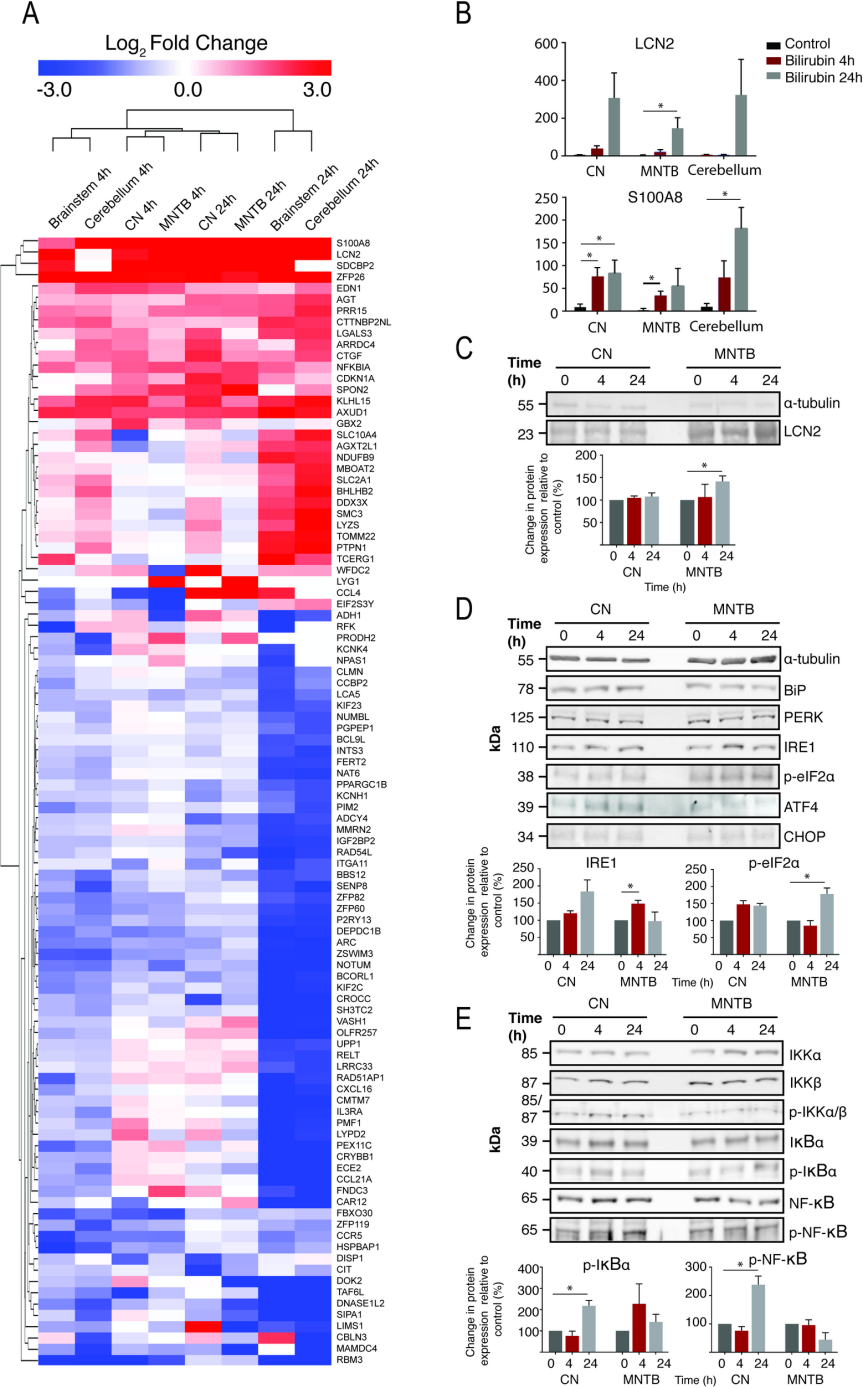
Bilirubin toxicity induces ER stress and inflammation in a novel model of bilirubin toxicity. **A)** Whole genome gene expression analysis was carried out on multiple brain regions following bilirubin exposure: brainstem, cerebellum, cochlear nucleus (CN) and the medial nucleus of the trapezoid body (MNTB). The top 100 genes with the greatest fold changes across all datasets are shown. Up-regulated genes are shown in red and down-regulated genes in blue. The values represent log_2_ fold change compared to control samples. Samples and genes were clustered using the Euclidean distance algorithm. Quantification of the two mRNAs most highly up-regulated in response to bilirubin (mean±SEM, n = 4, arbitrary fluorescence units) for **B**) lipocalin-2 (LCN2) and **C**) S100A8. Samples marked with * are significantly changed (p<0.05) following a Welch t-test. Representative immunoblots for **D**) ER stress and **E**) NF-kB pathway marker proteins in the CN and MNTB following bilirubin exposure for 4 or 24h (n = 3). Where significant changes in protein expression were detected by ANOVA, densitometry-based quantification of the immunoblots are included (*p<0.05).

Up-regulation of gene expression markers of ER stress (DUSP1, CTGF and EDN1) and the NF-κB pathway (JUN, NF-ΚBIA, and NR4A2) were observed in all of the samples and time points measured (Fig 3A). Transcriptional targets of NF-κB, such as NF-ΚBIB, FAS, BCL2L1 and CCL4, were clustered into two groups that were either increased across all brain regions and time points or were specifically up regulated in the CN and MNTB. There were also two large clusters of highly up- and down-regulated genes that were only present in the cerebellum and the brainstem at 24 hours. One of these up regulated genes, PTPN1, is also an NF-κB target gene. Two of the most striking up-regulated genes were S100A8 and LCN2, which code for a pro-inflammatory calcium binding protein S100-A8 (S100 calcium binding protein A8) and a pro-inflammatory cytokine and NF-κB target gene, respectively. Both genes showed a time-dependent up-regulation in the brainstem at all-time time points (Fig 3B and 3C). The increases in LCN2 were significant (p < 0.05) in the MNTB at 24h; while for S100A8 the up-regulation was significant (p < 0.05) in the CN at 4h and 24h, in the MNTB at 4h, and in the cerebellum at 24h. These results show that although the effect of bilirubin *in vivo* is more subtle than *in vitro* exposure, both ER stress and NF-κB pathway activation occur in the *in vivo* model.

To investigate further, immunoblots for markers of the NF-ĸB and ER stress pathways were carried out in microdissected CN and MNTB (Fig 3B). Immunoblotting confirmed the significantly increased expression of the NFκB-target gene and cytokine, LCN2, in the MNTB after 24h of bilirubin exposure, which corresponds to the increased expression observed in the microarray data. Immunoblots for protein markers of ER stress showed a significant increase in IRE1 at 4h and p-eif2a at 24h in the MNTB. Overall, the *in* vivo increases in ER stress markers were subtler than observed *in vitro*. Immunoblots for markers of the NFκB pathway showed few changes apart from a significant increase in p-IκBa and p-NFκB in the CN at 4h and 24h, respectively; similar to the results observed *in vitro*. Taken together, this further supports the hypothesis that bilirubin activates ER stress and NFκB pathways *in vivo* and increase the expression of pro-inflammatory cytokines, such as LCN2.

### Bilirubin induces a neuroinflammatory response similar to LPS

Based on the hypothesis that bilirubin triggers an inflammatory response in the brain, we compared the response with a known inducer of neuroinflammation: lipopolysaccharide (LPS). The NF-κB pathway is heavily involved in the inflammatory response, so we investigated bilirubin-induced inflammation in the brain by comparing it to the LPS model [29]. First, we compared the gene expression profiles induced by bilirubin and LPS; and then we tested whether LPS-induced inflammation had a deleterious effect on the auditory system.

**Fig 4:**
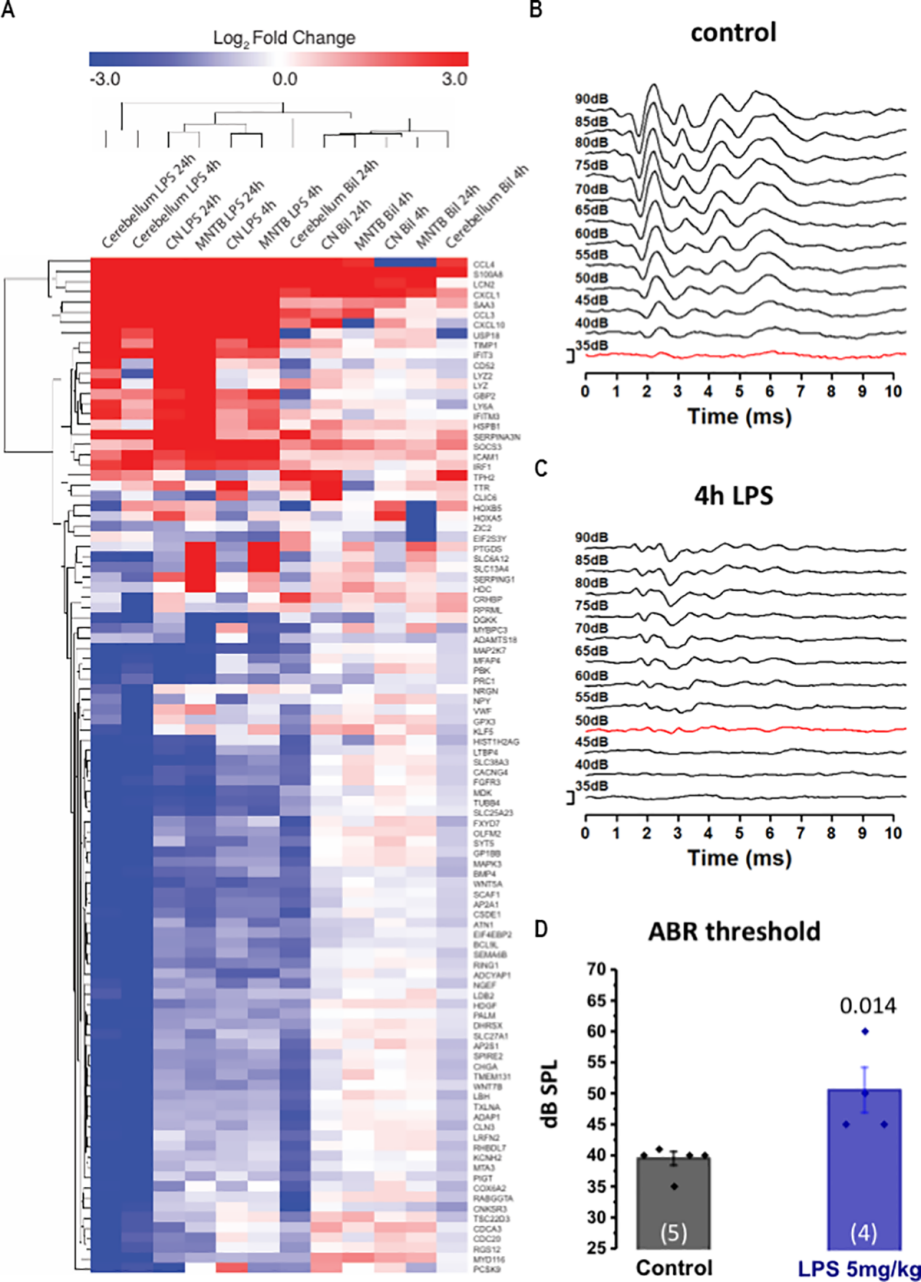
Lipopolysaccaride (LPS) a known neuro-inflammatory agent and bilirubin share significant similarities in their gene activation profile and both cause a rise in auditory threshold. **A)** Whole genome gene expression analysis was carried out on brain tissue following bilirubin or LPS exposure. The top 100 genes with the greatest fold changes across all datasets are shown. Up-regulated genes are shown in red and down-regulated genes in blue. The values represent log_2_ fold change compared to control samples (n = 4). Samples and genes were clustered using the Euclidean distance algorithm. **B)** The ABRs from a mouse are shown before LPS injection and **C)** 4h after LPS injection in the same animal. ABR responses are displayed over volume range of 35 to 90 dB (SPL), with the trace at threshold indicated in red. In this paired data LPS clearly raised auditory thresholds. **D)** Average data are plotted as Mean±SEM auditory thresholds, shown before and after LPS injection; LPS significantly increased auditory threshold (39±2 dB SPL for control n = 5 vs 50 dB ±7 dB SPL after LPS n = 4, p = 0.014, t test).

We employed intraperitoneal injection of LPS (5mg/kg) at a dose previously shown to induce neuroinflammation in mice [29]. Whole genome gene-expression analysis was used to compare the gene expression pattern (Fig 4A). There was a high degree of correlation between the gene expression pattern induced by bilirubin and that induced by LPS in all regions assayed, particularly in the cerebellum. Many of the most highly correlated genes were involved in inflammatory signalling; such as CCL3, CCL4, CXCL1, CXCL10, TIMP1 and SOCS3 and included LCN2 and S100A8. We also investigated the effect of LPS on hearing and the auditory brainstem. This showed a significant rise in the ABR threshold (from 39±2 dB SPL for control n = 5 to 50±7 dB SPL after LPS n = 4, P = 0.014, Fig 4B–4D) and corroborated previous data on the effect of LPS or bacterial infection on the auditory system in other animal models [39–42]. The rise in ABR threshold caused by LPS was smaller than that produced by bilirubin (compare Fig 2D with Fig 4C) but the broader similarity is consistent with bilirubin toxicity producing neuro-inflammation to which the auditory brainstem is particularly sensitive.

### Anti-inflammatory agents protect against bilirubin-induced auditory damage

Data from both the *in vitro* and *in vivo* models employed here suggest that the inflammatory response is a key process involved in bilirubin toxicity. We investigated this further by interference with the inflammatory process either genetically, using transgenic animals lacking LCN2, or pharmacologically using inhibitors of key inflammatory proteins, and measured the impact on hearing loss caused by bilirubin.

**Fig 5:**
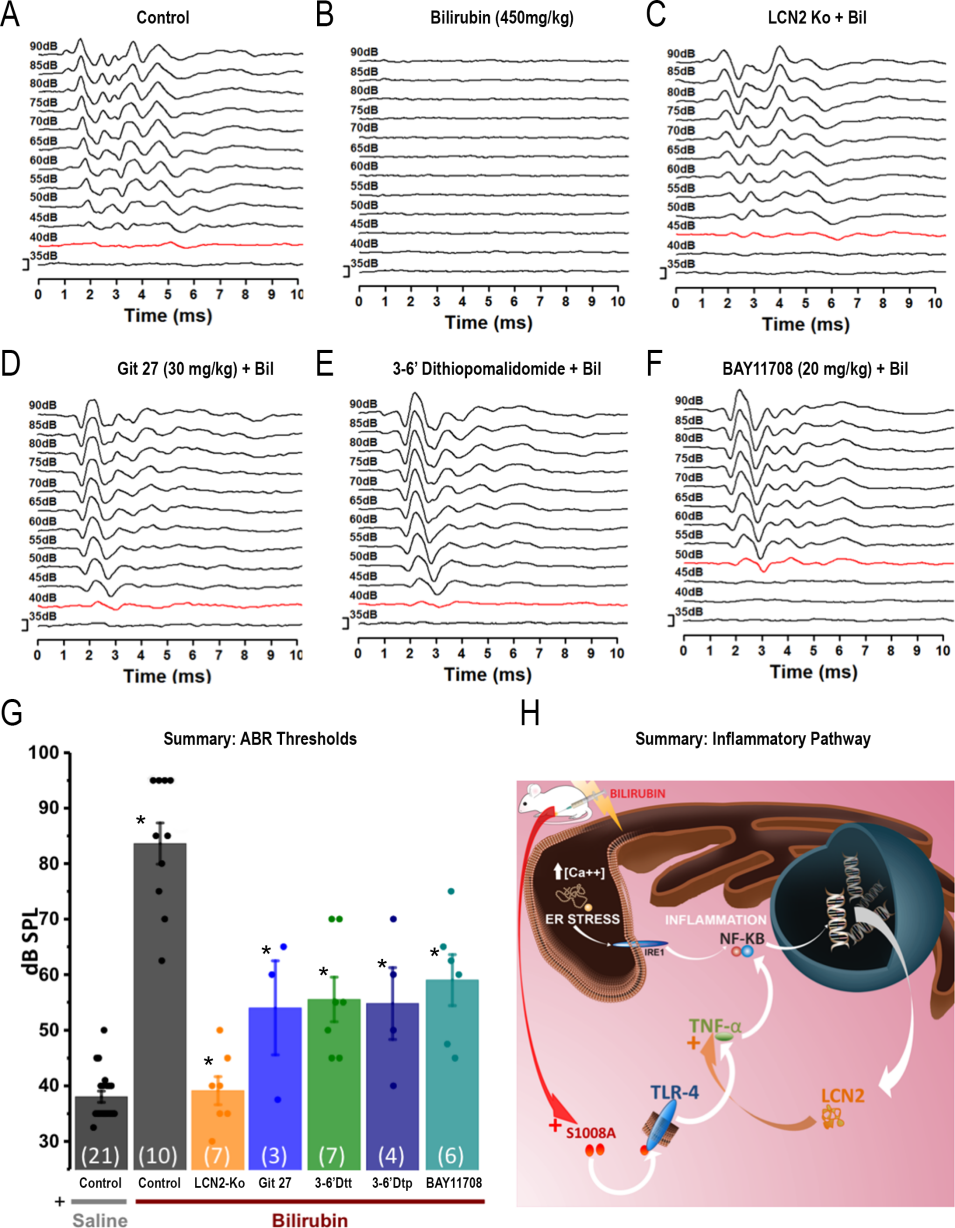
Suppression of neuroinflammation protects hearing from bilirubin toxicity. ABR measurements were taken following bilirubin exposure combined with genetic and pharmacological intervention in inflammatory signalling. **A-F)** Representative ABR traces for each condition are plotted across an intensity range of 35-90dB, traces in red indicate the threshold, scale bar is 1μV. a) Control; b) Control exposed to bilirubin alone; c) Lipocalin-2 (LCN2KO) knockout mouse. d) Git 27 (TLR inhibitor); e) 3,6’Dtt (novel TNF-α inhibitor). f) Bay 11708 (NF-κB inhibitor). **G**) ABR threshold summary data with mean ± SEM; statistical significance was assessed by ANOVA (followed by Bonferroni, see text for numerical values). Comparisons are as indicated by the star * (p<0.001) with comparison of:- saline control vs bilirubin control; and bilirubin control and each test condition. **H)** Summary diagram illustrating the overall conclusion that multiple pro-inflammatory pathways interact to induce inflammation following bilirubin exposure.

The inflammatory cytokine LCN2, was consistently up-regulated in the brain following bilirubin exposure. The hearing thresholds of homozygous LCN2 knockout mice (backcrossed onto a CBA/Ca background) were comparable to control WT CBA/Ca mice (WT mice: ABR mean threshold: 39 ± 0.7 dB SPL, n = 23 *versus* LCN2-/- KO mice: 40.8± 1.4 dB SPL, n = 12; t-test not significant p = 0.22). The hearing of LCN2 KO mice was protected from bilirubin toxicity. The ABR thresholds after bilirubin exposure were comparable to those of control animals (compare Fig 5A and 5C) and resistant to the hearing damage caused by bilirubin (Fig 5A–5C). A summary bar graph of the ABR thresholds is provided in Fig 5G. ANOVA analysis followed by Bonferroni correction confirmed that bilirubin significantly raised thresholds in WT mice, but not in LCN2 KO mice. WT mice receiving saline injections (sham) had thresholds of 38.1 ± 10 dB SPL n = 21, whereas WT mice injected with bilirubin had thresholds of 83.7±3.7 dB SPL (n = 10, Bonferroni P = 2.9E-16). In contrast LCN2 KO mice injected with bilirubin had thresholds of 39.2±2.5 dB SPL, which were not significantly different from control (n = 7, Bonferroni p = 1). Anti-inflammatory pharmacological agents were employed to test if they had a protective effect. Several different anti-inflammatory agents were administered *in vivo* by IP injection 30 min prior to bilirubin exposure, and then hearing was assessed using ABR after 4h (Fig 5D to 5F).

We detected mRNA for S100A8 as being highly upregulated following bilirubin exposure in multiple brain areas. S100A8 protein is pro-inflammatory and an endogenous ligand for Toll-like receptor 4 (TLR4), itself a mediator of inflammation by activating NF-κB and increasing TNF-α synthesis [43]. An antagonist of TLR4 was administered by intraperitoneal injection, Git27–30 mg/kg, 30 min before bilirubin injection [44] and this partially protected the ABR from bilirubin toxicity (the auditory threshold in mice pre-treated with Git27 was 54.1±8.4dB SPL n = 3; versus mice injected with bilirubin alone having a threshold of 83.7±3.7 dB SPL, n = 10; p = 1.9 E-04, statistically different with Bonferroni correction; Fig 5D and 5G). Several small molecule drugs are available that interfere with the synthesis of TNF-α, a major driver of the inflammatory response. Thalidomide derivatives are proposed as a new generation of anti-inflammatory molecules that act by destabilizing TNF-α mRNA [45]. We injected 3,6' dithiothalidomide (3–6’Dtt) or 3,6’ dithiopomalidomide (3–6’Dtp) (100mg/kg), 30 min prior to bilirubin injection and these mice exhibited an average auditory threshold of 55.7±3.9 dB SPL, n = 7; and 55±6.4 dB SPL, n = 4, respectively. This dose conferred significant protection compared to mice injected with bilirubin alone: which exhibited a hearing threshold of 83.7±3.7 dB SPL n = 10, (Bonferroni p = 1.8E-06 for 3,6' dithiothalidomide and p = 4.7E-05 for 3,6’ dithiopomalidomide; Fig 5E and 5G). Finally, we inhibited I*κ*B kinase (IKK), a signal-transducing protein in the NF-κB pathway, with BAY 11708 (20mg/kg) [46]. The auditory threshold following BAY 11708 was 59.1±4.5dB SPL, n = 6, which was significantly reduced compared to mice injected with bilirubin alone: 83.7±3.7 dB SPL n = 10, (Bonferroni p = 6.9E-05, Fig 5F and 5G).

Overall these results support the hypothesis that neuroinflammation is a major aspect of bilirubin toxicity and by interfering with various parts of the inhibitory signalling pathways we can ameliorate the deleterious effect of bilirubin on the auditory system (Fig 5H).

## Discussion

### A new mouse model of hyperbilirubinaemia

Hyperbilirubinaemia causes neurological and auditory deficits, particularly early in development. Here we have developed a new animal model of bilirubin toxicity and investigated the consequences of this toxicity in the auditory brainstem, which is associated with hearing loss. The most commonly used animal model of hyperbilirubinaemia is the Gunn rat and Ugt1^-/-^mice [24,47,48]. Gunn rats having been administered sulfadimethoxine (to displace endogenous bound bilirubin from albumin) have been used to investigate bilirubin toxicity [6,21,23]. However, the use of the genetic modified models have physiological and practical limitations. First, the acute hyperbilirubinaemia is induced on a background elevation of bilirubin. Despite the use of phototherapy to limit the accumulation of bilirubin and reduce further damage to the brain, it is possible that damage is also caused by bilirubin exposure prior the phototherapy. Therefore, the definition of the ‘control’ condition is relative to this background level of bilirubin insult. Second, after injection the animals do not recover. Third, in the case of the Gunn rat, the use of rats practically limits comparison with transgenic animal models to explore biological mechanisms. Therefore, we developed an acute–*in vivo* model of bilirubin toxicity in mice from which recovery is possible. We administered bilirubin by intraperitoneal injection along with sulfadimethoxine to minimise endogenous serum buffering. The dose of bilirubin and sulfadimethoxine was titrated using two key physiologically relevant metrics of bilirubin toxicity: movement impairment and auditory damage (Fig 2). At a dose of 450mg/kg bilirubin caused movement impairment (Fig 2A) and a profound increase in the auditory threshold at 4 hours (Fig 2D), from which recovery was observed by 24 hours (Fig 2E) and 5 days. This model has several benefits: first, it has a rapid onset and time-course and can be induced at any time of the animal development–at least up to P22. This is important for the study of the auditory system where hearing can be investigated only after opening of the auditory canal at P12. We can record ABR in control condition and after the exposure to bilirubin, in the same animal and over short a rapid time-course, which would be impossible to achieve using the chronic model. Also, the acute model developed in this paper can be applied to any mouse strain (including transgenic mice, used here and backcrossed onto CBA/Ca). Third, it is reversible, allowing dissection of biochemical and physiological changes associated with the acute toxicity of bilirubin.

### Bilirubin causes ER stress and NF-ĸB activation

We sought to gain an initial insight into bilirubin toxicity by exposing the neuroblastoma cell line, SHSY5Y, to bilirubin *in vitro*, *a*dopting a similar approach used by Calligaris et al 2009 [19]. In our work for the first time, whole genome gene expression analysis following bilirubin exposure was compared to the gene expression pattern produced by other chemicals contained within the cMap database. The most ‘similar’ chemical identified was thapsigargin (Fig 1A) which causes ER stress by inhibiting the SERCA ATPase proteins that maintain the high calcium concentration in the ER. Similarities to other chemicals known to induce ER stress were found and the gene expression pattern itself contained many transcriptional hallmarks of ER stress (Fig 1B). ER stress is a cellular process involved in detecting and clearing unfolded proteins from the ER. Misfolded proteins are detected by IRE1, PERK and ATF6 and increase the expression of chaperones such as BiP and DNAJB9, in order to aid protein folding. The total protein load is also reduced by attenuating translation via eIF2α [30,31,49]. When these measures fail, ER stress initiates apoptosis through the induction of CHOP and caspase activation [32,50]. ER stress has been identified in a number of pathological processes in the brain [33]. We also detected several mRNA changes indicative of NF-ĸB pathway activation (that were not previously detected in similar studies) [19], which is activated in response to cellular stress and can trigger the inflammatory response [34]. For this reason, we immunoblotted for protein markers of both the ER stress and NF-ĸB pathways (Fig 1C and 1D). The immunoblots confirmed the increase in expression of multiple ER stress marker proteins, as well as an increase in the active phosphorylated form of NF-ĸB. However, there were no changes in other upstream proteins associated with NF-ĸB activation. This suggests that the NF-ĸB pathway was not activated in a canonical fashion and coincided with a distinct change in ER morphology, similar to that observed with thapsigargin (Fig 1E). We conclude that by using unbiased methods to determine the most prominent biochemical events following acute bilirubin exposure *in vitro*, we have detected activation of both the ER stress and NF-ĸB pathways.

We then applied the same assays to assess the biochemical events that occur in our mouse model of hyperbilirubinaemia. We anticipated that the complex structure and multiple cell types in brain tissue would complicate the gene- and protein-expression profiles, but microarray analysis nevertheless detected several transcriptional markers of ER stress and a large enrichment of transcripts for proteins either involved in or downstream of the NF-ĸB pathway (Fig 3A), including the consistently highly upregulated transcripts for S100A8 and LCN2, the latter of which we confirmed by immunoblot (Fig 3D). The gene expression profiles also showed a difference in transcriptional response between the larger anatomical regions (brainstem and cerebellum) compared to the CN and MNTB, although the response of the CN and MNTB appeared similar. We carried out immunoblots on these two key structures of the auditory pathway to investigate protein-level events associated with bilirubin toxicity. We did not detect any markers of ER stress in the CN, but detected increases in both IRE1 and p-eIF2a at 4h and 24h, respectively. Conversely, we detected no changes in NF-ĸB markers in the MNTB, but detected increases p-IkBa and NF-ĸB at 4h and 24h, respectively in the CN. Interestingly, ER stress has been shown to activate the NF-ĸB pathway independently of canonical upstream signalling proteins [51]. During ER stress, IRE1 has been shown to maintain levels of IKK, causing an increase in p-IkBa, resulting in the phosphorylation and subsequent activation of NF-ĸB. This results in the translocation of NF-ĸB to the nucleus and the transcription of NF-ĸB-target genes, several of which we detected in both in the CN and MNTB. Notably, the NF-ĸB target genes are more highly up-regulated in the CN with increased protein levels of NF-ĸB-pathway marker proteins. Therefore, we postulate that bilirubin initiates an ER-stress response in the auditory brainstem, activating the NF-κB pathway, causing a broad inflammatory response.

Based on the above findings we compared the gene-expression pattern induced by bilirubin with that of LPS which is a well-established model of neuroinflammation. There was a high degree of correlation in the genes most up-regulated by bilirubin and LPS, which included the consistently highly up-regulated genes S100A8 and LCN2, along with CCL4, CCL3, CXCL1 and CXCL10 –all of which are inflammatory chemokines involved in inflammatory signalling [52] (Fig 4A). LPS also increased the auditory threshold at 4h, albeit to a lesser degree than bilirubin. These data confirmed that bilirubin causes neuroinflammation and that the neuroinflammatory process can perturb auditory function.

### Interfering with the bilirubin-induced inflammation protects hearing

As bilirubin caused neuroinflammation in our mouse model of hyperbilirubinaemia, we postulated that interfering with the inflammatory process would protect hearing. NF-ĸB pathway activation produces pro-inflammatory cytokines which perpetuate and amplify the inflammatory process. The calcium binding protein S100A8 was detected as highly up-regulated by bilirubin and LPS in our *in vivo* experiments and in previous experiments [53]. S100A8 is the endogenous ligand for Toll like receptor 4 (TLR4) which activates NF-ĸB [44], whereas LCN2 is an NF-ĸB target gene and pro-inflammatory cytokine, that can itself activate the NF-ĸB pathway and is implicated in inflammation [54], neurodegeneration [55] and noise-induced hearing loss [56]. Finally, TNF-α is a key target gene of NF-ĸB and a well-established activator of the NF-ĸB pathway and inflammatory mediator [57]. Although not detected as transcriptionally up-regulated here, its importance as an inflammatory mediator suggested that inhibitors of TNF-α generation (thalidomide derivatives) [45] along with a TLR4 inhibitor (Git 27) [44], an NF-ĸB inhibitor (BAY 117084) [46], and LCN2 null mice could be employed to determine if suppression of these pro-inflammatory mediators were protective. Each showed a significant protection from bilirubin toxicity (as shown in Fig 5), supporting the hypothesis that NF-ĸB pathway activation and inflammation are key processes of bilirubin-induced hearing loss and providing insights into possible treatments for hyperbilirubinaemia. Our results showed the involvement of Nf-Kβ and TNF-α similar to that observed in a transgenic mouse model for Jaundice [47]. There is evidence that meningitis [58] and cytomegalovirus infections [59] are associated with activation of inflammatory mediators and cause hearing loss. Local inflammation following noise-induced hearing loss [60] is also reported and indeed it seems inevitable that local and systemic inflammatory mediators could interact to exacerbate pathological consequences.

In previous studies of bilirubin exposure in the Gunn rat we have shown that the calyx of Held synapse is destroyed by an acute increase in serum bilirubin [23], but in that model, bilirubin has also been chronically elevated due to lower excretion efficiency from birth (due to poor conjugation by uridine-diphosphoglucuronyl-tranferase in the liver). In this mouse model of bilirubin toxicity the elevated bilirubin is highly acute and is induced in wildtype mice, which after the initial insult can rapidly remove the excess bilirubin through their normal metabolism. The question of how bilirubin achieves the transient loss of hearing associated with this ER Stress and inflammation is an important open question, which requires further study. The fast recovery period strongly implies that the giant synapses are not destroyed in the mouse model (in contrast to the Gunn rat). Future electrophysiological studies will investigate bilirubin-induced changes in synaptic function and neuronal excitability changes in order to identify the cellular mechanisms of this toxicity. Here we have clearly demonstrated a transient damage to the auditory pathway caused by bilirubin, but this differes from what has been observed in the Gunn rat model. The broader implications of jaundice and the potential for compromising brain function during development are receiving increased attention. Neonatal hyperbilirubinaemia [61] has been associated with auditory processing disorders in a recent 30 year prospective study which reported long-term negative consequences and cognitive abnormalities in humans and evidence for auditory neuropathy and dysynchrony [6].

### Conclusions

This work presents a new mouse model of acute hyperbilirubinaemia, which displays the major symptoms observed in humans of movement impairment and temporary hearing loss, and which is also reversible. We characterised the model extensively using unbiased analyses to detect the biochemical changes that occur in structures of the auditory pathway, identifying ER stress, NF-ĸB-pathway activation and inflammation as key mechanisms of bilirubin toxicity. Suppression of the inflammatory response, protected against hearing loss and highlighted exciting new options for therapeutic targets. This work also serves as a proof of principle for rapid *in vivo* drug screening for drugs that mitigate hyperbilirubinaemia and neuro-inflammation. Since many other neurodegenerative disorders also cause neuro-inflammation [62,63] this mouse model could be used to screen potential therapeutics much more rapidly than other slowly developing models of neurodegeneration. One of the unique features of this model is its reversibility. This mouse model offers the means to investigate the long-term neurological damage caused by hyperbilirubinaemia.

## Material and methods

### Cell culture

SHSY5Y cells were obtain from ATCC (Manassas, VA) and maintained in DMEM-F12 supplemented with 10% fetal bovine serum (FBS). All cells were passaged at 90% confluency and experiments only carried out on cells with a passage number below 10. The cells were exposed to bilirubin when at approximately 80% confluency. Bilirubin powder Sigma (143701G) was dissolved to have a final concentration of 50μM in media (equivalent to 6.39μM of free bilirubin) measured using a commercial kit BXC0193B Fortress diagnostics).

### Animals

All animal experiments were performed under a Project License and in accordance with the UK Animals (Scientific Procedures) Acts of 1986 and with the approval of local ethical committees by personnel that had undergone special training and possessed a UK Government Personal License to handle and conduct animal experiments. Animals were housed in the Central Research Facility at the University of Leicester and provided with free access to food and water, monitored daily. Both male and female mice were used (with the control, background strain being CBA/Ca). We compared the behavioural impairment (see below) in CBA/Ca mice at 4h after IP bilirubin injection at ages P11 ±1 or P19 ±2. The P11 animals were no more vulnerable, in that the mean ataxia score were similar to P19 and were not statistically significant: P11 (N = 15) score 2.28±0.2; *P*19 (*N* = 8) score 2.07±0.2; t = 0.29, not significant. The microarray experiments were conducted in P11 animals while the hearing damage was assessed in P19 mice, after the opening of the auditory canal. We have also injected mature animals, older than 6 months via IP injection (450mg/kg bilirubin+300mg/kg Sulfadimethoxine, n = 2); or via intravenus injection (n = 2) but these mature animals were resistant to bilirubin toxicity, consistent with the known sensitivity of young animals to bilirubin.

### Assessment of the behavioural impact of bilirubin injection and experimental humane end-point

A behavioural Index of movement impairment and well-being was developed to test for bilirubin toxicity in which dosed animals were scored from 0 to 3, based on both movement and well-being of the individual animal. Score ‘0’ = animal appears normal; ‘1’ = animal is less explorative than an untreated mouse; ‘2’ = animal first starts to exhibit a mild ataxia. Experiments were conducted when animals had reached this stage. A score of ‘3’ = animals tend not to move spontaneously or move slowly. Animal behaviour was scored at 30 minutes and every hour for the first 6hr after treatment, then at 24hr. The humane end point was defined so that animals that scored above 2 by 4hr after treatment were culled. Of 35 mice receiving bilirubin treatment and monitored for 24hr, 7 were culled (at 4hr) and 2 died; this was the end of the experiment for 16 mice and they were humanely killed; 6 of these mice were monitored for 5 days of recovery, no further deaths were observed and these animals were humanely killed after their ABRs had been measured. Four animals received treatment with LPS, all were humanely killed at 24hr and none had died. Seven LCN2 knockout mice received bilirubin treatment (Fig 5G), all were humanely killed at 24hr and none had died. Twenty mice received bilirubin treatment plus anti-inflammatory drug treatment (Fig 5G), all were humanely killed at 4hr and none had died.

### Preparation of bilirubin and sulfadimethoxine solution and dosing

A combined intraperitoneal injection of Bilirubin (Sigma, 143701G) and Sulfadimethoxine sodium salt (S7385-10G) solution was prepared, as below and administered to CBA/Ca mice at ages of P11 ±1 (11 days after birth) or P19±2 (for ABRs measure) at the dosage of 450mg/kg bilirubin+ 300mg/kg Sulfadimethoxine, unless otherwise stated in the text. Throughout this manuscript this combined injection is referred to as ‘bilirubin’. Bilirubin was dissolved in NaOH (0.5 M) Sigma (S8045-50MG) with the pH adjusted to 8.4 with HCl; Sulfadimethoxine sodium salt (S7385-10G) was then added and the pH again checked (Note: a pH lower than 8.4 caused precipitation of bilirubin). This procedure was performed under dim light.

### Thalidomides analogues emulsion for injection

Thalidomide analogs were synthesized to >99% purity and their structure was confirmed by ^1^H NMR and mass spectrum analyses. Predetermined quantities of drug were weighed out into glass vials (SIGMA, cat # Z256064-1PAK), and 10–20 glass beads (2 mm diameter) were added to finely mill the compounds during a 10 minute vortex. A desired volume of vehicle (1% carboxymethyl cellulose–CMC- sodium salt in filtered sterilized saline 0.9%) was then added. A uniform suspension was prepared by vortexing for a minimum of 10 minutes, and this suspension was IP injected, with constant use of the vortex between injections to maintain the suspension. LPS (Sigma Cat. L5293; 5mg/kg) was dissolved in saline and injected IP.

### Hearing test: Auditory brainstem response

CBA/Ca mice were anaesthetised with a combination of fentanyl (0.15mg/kg), fluanisone (5mg/kg) and hypnovel 2.5mg/kg) intraperitoneally. ABRs were recorded as previously reported [64, 65]. ABR recordings were made in control conditions and at 4, 24, or occasionally as indicated 5 days after bilirubin injection. Note that bilirubin-injected animals were more sensitive to anaesthetic at 4 h after injection, but no difference was observed at 24h.

The ABRs were measured on two different sets of equipment. The measurements (shown in Fig 2) were recorded as described in reference 64. ABRs were evoked by clicks (broadband between 2 and 20 kHz,100 *μ*s), which were produced by a Thurlby Thandar arbitrary waveform generator (TGA 1230, 300 MHz, Tucker Davis, Alachua, FL, USA) and applied at 10 Hz unilaterally in free field using a B&K microphone (B&K 4192). The final ABR constituted an average of 100–400 individual traces recorded by intradermal electrodes (positive, negative and ground electrodes were inserted subcutaneously at the vertex, mastoid and back, respectively) with an input gain of 5000 connected to an amplifier (Medelc Sapphire 2A, Oxford Instruments, Oxford, UK) and sampled at 16 kHz. Hearing thresholds were determined by attenuating the initial stimulus intensity by 10 dB (Tucker Davis) until ABR waves I and II could no longer be visually defined.

Different equipment was employed for auditory testing in Figs 4 and 5, as described in in reference 65. Recordings was made within a sound attenuating chamber (IAC Ltd, Mini-Acoustic Chamber MAC1). Needle electrodes were placed subcutaneously around the mouse's head; a ground electrode over the right bulla, a reference electrode over the left bulla and an active electrode on the vertex. Placement was in a natural prone position facing a loudspeaker, at a distance of 20 cm from the leading edge of the speaker to the animal's interaural axis. A custom software application, driving Tucker Davis Technologies (TDT) System 3 hardware (RP2.1, RA16, PA5) was used for acoustic calibration, presentation of stimuli and recording of evoked potential responses. Stimuli were calibrated using a PCB Piezotronics Inc. microphone system (Model 378C01 condenser microphone, Model 426B03 preamplifier and Model 480C02 signal conditioner) and presented in dB SPL re. 20 mPa. Stimuli consisted of clicks (10 ms duration). These were generated at a sample rate of 97.656 kHz, converted to the analogue domain (TDT RP2.1), attenuated to achieve the desired final sound level (TDT PA5), amplified (TDT SA1) and presented as groups of 256 stimuli at 42.6/sec with 5 dB increments from 0 to 95 dB SPL, via a CTS Type 241 transducer (RS components). Evoked potentials detected by the subcutaneous electrodes in response to the stimuli were amplified and digitized (TDT RA4LI head stage and RA4PA preamplifier) before being digitally filtered (TDT RA16; 300e3000 Hz) further amplified and stored in an averaging buffer at a sample rate of 97.656 kHz. The auditory brainstem response (ABR) was saved as the average response to 256 presentations of each acoustic stimulus. The threshold determination was purely visual. ABR thresholds for each click were estimated from the data recorded as the lowest sound level that evoked a recognizable portion of the overall ABR waveform by visual inspection of responses stacked according to sound. If in doubt between 2 traces, the mid-value was used (ie. the value of 47.5 was chosen between 45 and 50 dB). For ethical reasons the maximal sound intensity was restricted to 95 dB, so note that this could have resulted in an underestimation of the most severe bilirubin auditory damage.

### Tissue collection for microarray and immunoblotting

For studies of brainstem and cerebellar brain regions, mice were killed by decapitation. The brain was removed, the brainstem and cerebellum dissected and snap frozen on dry ice for immunoblotting or placed in TRI reagent (Sigma Aldrich, UK) for RNA extraction. Illumina TotalPrep RNA amplification kit (Ambion, UK) and Illumina MouseRef-8 microarray chips (Illumina, USA) were used for microarray.

### Immunoblots

Immunoblots were carried out according to standard procedures as previously described [36]. The ER stress antibody kit (9956) and NF-ĸB antibody kit (9936) were obtained from Cell Signalling. The LCN2 antibody was obtained from Millipore (AB2267).

### Immunocytochemstry

SHSY5Y grown on 13mm coverslips cells were washed with PBS and fixed with 4% paraformaldegyde. The samples were permeabilized with PBS Triton X-100 (0.1%). Antibodies were prepared in 3% BSA. The calreticulin antibody was obtained from Abcam (AB2907) and the Goat Anti-chicken secondary was obtained from Invitrogen. The cover slips were mounted using DAPI-containing mounting media (Invitrogen) on pre-cleaned microscope slides and visualized on a Zeiss Axioscope confocal microscope.

### RNA extraction and microarray analysis

RNA extraction was carried out using a modified form of the phenol-chloroform method and prepared for microarray analysis using the Illumina TotalPrep RNA amplification kit (Ambion, UK) as previously described [28,36,38,66]. The labelled cRNA samples were assayed using Illumina MouseRef-8 microarray chips (Illumina, USA).

### Analysis

Microarray data were normalised by mean scaling and compared by Welch t-test using ArrayTrack [67,68] software Gene expression trends were visualised using heat maps hierarchically clustered using a ‘city-block distance’ algorithm using Gene-e software [69]. For microarray analysis and immunoblotting, tissue was collected from 4 animals for each experimental condition.

Densitometry was used to quantify Immunoblots using ImageJ software. Following normalization to control samples and loading controls, statistical analyses (ANOVA) were carried out using GraphPad Prism software.

For ABR analysis GraphPad prism 6 (GraphPad software, Inc.), Origin 8 (Origin lab corporation) were used. For behaviour and ABRs the number of animals used (n) is specified in the legend or in the figure. For Behavioural studies: the data for dose-response relationships were fitted with the following Hill equation y = A1 + (A2-A1)/(1 + 10^((LOGx0-x)*p)) were A1 = 0 and A2 = 3, LOGx0-x = dose to have 50% of maximum effect and p = slope hill. For investigating the statistical significance at the level of 95% in the experiments of ABR and pharmacology, t-test or ANOVA (post Bonferroni) tests were used, as specified in the text. Data in the text are reported as mean ± SEM, n = number of animal used for the statistics and p value.

## Supporting information

S1 File Behavioral manifestation of bilirubin toxicity at 4h after injection. The following video shows the behavior of a mouse in injected with saline (control, no tail mark) and litter-mates injected with bilirubin 450mg/kg 4h before the video was recorded. The latter (3 tail marks) is in the early stage 2. All of the animals used for our experiments were in this stage. Mice with 1 and 2 tail marks were in late stage 2.
(MP4)

S2 File Humane endpoints checklist. (DOCX)

## Abbreviations

ABR: auditory brainstem response
BBB: Blood-Brain-Barrier
BIND: Bilirubin-Induced Neurological Dysfunction
cMap: Connectivity Map
CN: Cochlear Nucleus
eIF2α: Eukaryotic translation initiation factor-2α
ER: endoplasmic reticulum
IP: intraperitoneal
IRE1α: Inositol-requiring enzyme-1
LCN2: lipocalin-2
LPS: lipopolysaccharide
MNTB: Medial Nuclei of the Trapezoid Body
NF-κB: nuclear factor kappa-light-chain-enhancer of activated B cells
S100-A8: S100 calcium binding protein A8
SERCA: sarco/endoplasmic reticulum calcium ATPase
TLR4: Toll-like receptor 4
TNF-α: Tumor necrosis factor alpha
UPR: unfolded protein response
