# Supplementary material for: Neuroinflammation and ER-stress are key mechanisms of acute bilirubin toxicity and hearing loss in a mouse model
Source: PLoS One. 2018 Aug 14;13(8):e0201022. doi: 10.1371/journal.pone.0201022 (PMC6091913; doi:10.1371/journal.pone.0201022)
Supplement: S2 File — (DOCX) [file pone.0201022.s002.docx]

*PLOS ONE* Humane Endpoints Checklist

*PLOS ONE* manuscript number: __PONE-D-18-036R1_____________________

**Complete the following if your study design includes death of a regulated animal as a likely outcome or planned experimental endpoint. Please also include all information in the Methods section of your manuscript.**

**ITEM 1.** **Describe whether humane endpoints* were used for all animals involved in the study.**

|  | **Recommendation** | **Section/Paragraph** |
| --- | --- | --- |
| **If humane endpoints* were used, report the following:** | | |
| **1** | **The specific criteria used to determine when animals should be euthanized** | **When the behavioural score exceeded 2, 4hr after treatment** |
| **2** | **Once animals reached endpoint criteria, the amount of time elapsed before euthanasia** | **The animals were anaesthetized at 4 hrs to test their hearing (ABR) and humanely killed at the end of this test, i.e. within 30 minutes of 4hrs** |
| **3** | **Whether any animals died before meeting criteria for euthanasia** | **2 animals died before meeting the criteria** |
| **If humane endpoints* were not used, report the following:** | | |
| **1** | **A scientific and ethical justification for the study design, including the reasons why humane endpoints could not be used, and discussion of alternatives that were considered but could not be used** |  |
| **2** | **Whether the institutional animal ethics committee specifically reviewed and approved the anticipated mortality in the study design** |  |

**ITEM 2.** **Include the following details of the study design and outcomes.**

|  | **Recommendation** | **Section/Paragraph** |
| --- | --- | --- |
| **1** | **The duration of the experiment** | **Lines 536 -548** |
| **2** | **The numbers of animals used, euthanized, and found dead (if any); the cause of death for all animals** | **Lines 520-531** |
| **3** | **How frequently animal health and behavior were monitored** | **541-544** |
| **4** | **All animal welfare considerations taken, including efforts to minimize suffering and distress, use of analgesics or anaesthetics, or special housing conditions** | **540-550** |
| **5** | **Any special training in animal care or handling provided for research staff** | **Lines 520-525** |

***Definition of a humane endpoint**

A humane endpoint is an experimental endpoint at which animals are euthanized when they display early markers associated with death or poor prognosis of quality of life, or specific signs of severe suffering or distress. Humane endpoints are used as an alternative to allowing such conditions to continue or progress to death following the experimental intervention (“death as an endpoint”), or only euthanizing animals at the end of an experiment. Before a study begins, researchers define the practical observations or measurements that will be used during the study to recognize a humane endpoint, based on anticipated clinical, physiological, and behavioral signs. These may include, for instance, body temperature or weight changes, tumor size or appearance, abnormal behaviors, pathological changes, ruffled fur, reduced mobility, body posture, or expression of specific body fluid markers. Please see the NC3Rs guidelines for more information.

**ARRIVE Guidelines**

*PLOS ONE* encourages authors to follow the [Animal Research: Reporting of In Vivo Experiments (ARRIVE) guidelines](http://www.nc3rs.org.uk/arrive-guidelines) for all submissions describing laboratory-based animal research and to upload a completed [ARRIVE Guidelines Checklist](http://www.nc3rs.org.uk/sites/default/files/documents/Guidelines/NC3Rs%20ARRIVE%20Guidelines%20Checklist%20%28fillable%29.pdf) to be published as supporting information.
